# Dielectric Response Spectroscopy as Means to Investigate Interfacial Effects for Ultra-Thin Film Polymer-Based High NA EUV Lithography

**DOI:** 10.3390/polym12122971

**Published:** 2020-12-12

**Authors:** Joren Severi, Danilo De Simone, Stefan De Gendt

**Affiliations:** 1 Department of Chemistry, KU Leuven, Celestijnenlaan 200F, B-3001 Leuven, Belgium; Stefan.DeGendt@imec.be; 2 Department of Advanced Patterning, imec, Kapeldreef 75, B-3001 Leuven, Belgium; Danilo.DeSimone@imec.be

**Keywords:** dielectric response spectroscopy, glass transition temperature, High NA EUV lithography

## Abstract

Extreme ultra-violet lithography (EUVL) is the leading-edge technology to produce advanced nanoelectronics. The further development of EUVL is heavily based on implementing the so-called high numerical aperture (NA) EUVL, which will enable even smaller pitches up to 8 nm half pitch (HP). In anticipation of this high NA technology, it is crucial to assess the readiness of the current resist materials for the high NA regime to comply with the demanding requirements of resolution, line-edge roughness, and sensitivity (RLS). The achievable tighter pitches require lower film thicknesses for both resist and underlying transfer layers. A concern that is tied to the thinning down is the potential change in resist properties and behavior due to the interaction with the underlayer. To increase the fundamental understanding of ultra-thin films for high NA EUVL, a method to investigate the interplay of reduced film thickness and different patterning-relevant underlayers is developed by looking at the glass transition temperature (T_g_) of polymer-based resists. To minimize the ambiguity of the results due to resist additives (i.e., photoacid generator (PAG) and quencher), it was opted to move forward with polymer-only samples, the main component of the resist, at this stage of the investigation. By using dielectric response spectroscopy, the results obtained show that changing the protection group of the polymer, as well as altering the polymer film thickness impacts the dynamics of the polymer mobility, which can be assessed through the T_g_ of the system. Unexpectedly, changing the underlayer did not result in a clear change in the polymer mobility at the tested film thicknesses.

## 1. Introduction

Slowly but steadily, extreme ultra-violet lithography (EUVL) is gaining ground in the high-volume manufacturing (HVM) of the current foundry technology node (N7), by companies such as Samsung and Taiwan Semiconductor Manufacturing Company (TSMC) [1,2]. Today, research is focused on enabling the future foundry nodes (N5, N3, and beyond) by extending beyond the use of EUVL. While 13 nm half pitch (HP) is the theoretical limit of EUVL today, the current state allows properly printing feature sizes down to 16 nm HP where considerable effort is put to enable acceptable defect levels. To advance from N5 to the future foundry technology nodes, a further reduction in feature sizes is again required. This further development of EUVL is heavily based on fabricating an optical system that can support a larger numerical aperture (NA), i.e., a larger lens system, in the so-called high NA EUVL [3]. This system, with an increase in NA value of 0.33 to 0.55, can capture diffraction orders over a larger area. Since the diffraction orders of smaller pitches are further apart from each other, having a larger lens enables printing smaller features. The introduction of this tool in the lab environment is envisioned for 2023 and is expected to be able to print down to 8 nm HP. In anticipation of the high NA technology, the focus needs to be on other challenges that may emerge when scaling the EUVL technology.

The resist has an important part to play in every lithography process by indirectly transferring the information present on the mask to the substrate when irradiated with light [4]. Throughout the optimization of lithographic technologies, a continuous downscaling of the thickness of the resist and underlayers can be seen. The reason for this is mainly process-related to prevent large aspect ratios (resist line height-to-width) when progressing to smaller features sizes. These large aspect ratios would otherwise cause pattern collapse during the development of the resist due to capillary forces during the rinse process [5]. This is also why the resist is then transferred into the underlayer, which is more etch resistant and rigid, enabling higher aspect ratios and better control of profile in this layer [6]. Furthermore, since EUV photons are ≈14 times as energetic as the previous 193 nm photons, a higher absorption is needed to capture as many photons (i.e., information) as possible. Due to the higher absorption, the photon absorption in these high-absorption materials can only be kept homogenous by reducing the film thickness. Currently, one of the major roadblocks is to find a resist that can resolve these material and process-related problems associated with the small feature sizes envisioned for high NA EUVL. 

As a result of the continuous downscaling, both resist film and underlayer film thicknesses are approaching the ultra-thin film regime, at or beyond the physical limits of bulk-phase behavior. This means that the interface interactions between the different layers (e.g., resist and underlayer) become increasingly dominant in ultra-thin films (≤30 nm), possibly changing material characteristics and thus making it challenging for the formulation of the resist to obtain good patterning performance on (different) underlayers. These changes include changes in dose to size (DtS), resist nanopattern profile, and amount of nanofailures, as well as inducing chemical inhomogeneity of the resist additives causing pattern degradation [7,8]. Therefore, an in-depth characterization of these properties versus film thickness is necessary to detect any future possible issues of the current patterning materials. The glass transition temperature (T_g_) of a chemically amplified resist (CAR) is an important property that relates to the post-exposure bake (PEB) temperature and influences acid diffusion, and thus also the resulting critical dimension (CD) and line-edge roughness (LER) of the resist line. Moreover, it influences the propensity to form defects (e.g., bridges or breaks) as well [9]. While it is apparent that some inherent characteristics of a polymer such as intramolecular and intermolecular interactions can influence the T_g_, it was already proven that the T_g_ depends on film thickness and interfacial interactions [10]. The origin of this discrepancy with the bulk T_g_ is believed to be an altered mobility of polymer chains at the interfacial layer through these interfacial interactions [11]. Therefore, this work uses T_g_ measurements as a metric to evaluate if polymer films at high NA film thicknesses are experiencing an increased effect from confinement and underlayer interaction.

In an initial effort to increase the fundamental understanding of ultra-thin films for EUVL, a relevant polymer of an open chemistry CAR platform (poly(hydroxystyrene-random-methyl methacrylate, P(HS-r-MMA)) is used to study the influence of the chemical structure of the protecting group, film thickness, and underlayer on the glass transition temperature of a polymer thin film. A methodology for dielectric response spectroscopy to extract T_g_ and use it for this parameter study will be given. The results from this study will be used to find its correlation to the resist patterning performance of the respective full resist formulations on a full field ASML NXE:3300B EUV scanner.

## 2. Materials and Methods 

### 2.1. Materials

**Figure 1:**
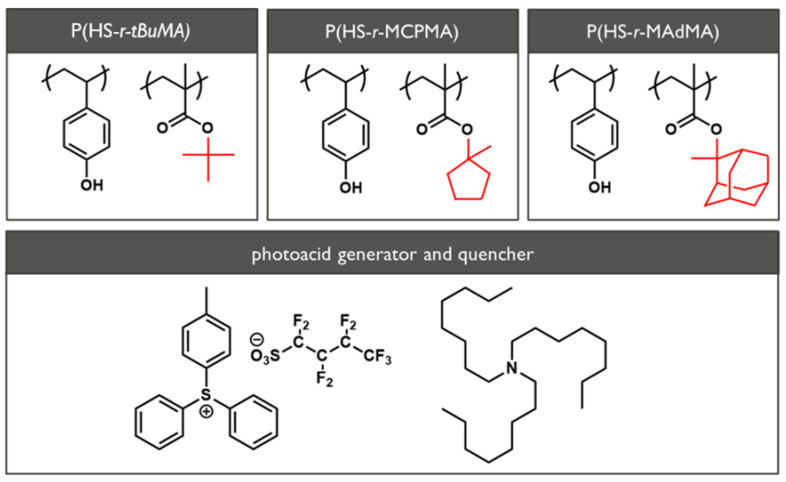
The chemical structure of the poly(hydroxystyrene-*ran*-tert-butyl methacrylate) (P(HS-*r*-tBuMA)), poly(hydroxystyrene-*ran*-1-methylcyclopentyl methacrylate) (P(HS-*r*-MCPMA)), and poly(hydroxystyrene-*ran*-1-methyladamantyl methacrylate) (P(HS-*r*-MAdMA)) polymers, and associated photoacid generator ((4-methylphenyl)diphenylsulfonium nonaflate) and quencher (trioctylamine) used in the full resist formulation.

Three polymers based on the poly(hydroxystyrene-*ran*-methyl methacrylate) (P(HS-*r*-MMA) platform with different protection groups, i.e., poly(hydroxystyrene-*ran*-tert-butyl methacrylate) (P(HS-*r*-tBuMA), poly(hydroxystyrene-*r*-1-methylcyclopentyl methacrylate) (P(HS-*r*-MCPMA), and poly(hydroxystyrene-*ran*-1-methyladamantyl methacrylate) (P(HS-*r*-MAdMA) were provided by FUJIFILM Electronic Materials (Yokohama, Japan). The molecular weight of the materials was determined to be 6887, 7007, and 8084 g/mol and the polymers consist of 53, 49, and 46 monomers respectively, with a 50:50 ratio between the two monomer units. The casting solvent used for spin coating was a mixture of PGMEA:PGMA with a respective ratio of 80:30. The chemical structure of the polymers can be found in Figure 1, where the differences between the polymers is indicated in a red color.

### 2.2. Methods

#### 2.2.1. Differential Scanning Calorimetry

Differential scanning calorimetry (DSC) is used to determine the T_g_ of bulk material in this study. It is based on the principle that when a sample undergoes a physical change, such as a transition from a rigid to a rubbery phase, a difference in energy compared to a reference cell is detected. By tracking these differences over a range of temperatures, it is possible to determine the T_g_ of the system. While it is a useful technique for bulk materials, it is much more difficult to apply this technique to polymer thin films. For this reason, it was opted to use dielectric response spectroscopy (DRS) to overcome this issue, and it is suited for polymer thin films. In the next section, a description of the DRS technique will be given.

#### 2.2.2. Dielectric Response Spectroscopy

Polymer chain motion, which is important in the determination of the T_g_, occurs at very short timescales, which makes it difficult to measure them directly. However, the introduction of the fluctuation dissipation theorem (FDT) of Callen and Welton allows investigating a macroscopic response of a system and correlating this with microscopic dynamics, since it allows correlating the macroscopic response to a small outer force to the spontaneous fluctuations of a system [12]. In dielectric response spectroscopy (DRS), the outer force is represented by a sinusoidal alternating electric field, which induces an oscillatory deformation in the sample. The sample that is measured will behave as a resistor capacitor (RC) circuit and under the influence of the applied frequency of the electrical field produce its own field because of the polarization of the sample. The generated field of the sample will not be instant or at the same level of the original field, i.e., it will display a phase lag and amplitude loss. By monitoring the dielectric loss (ɛ”) of the sample through a frequency range (10−^1^–10^7^ Hz), the obtained characteristic α-peak of the dielectric spectrum can be fitted for that specific temperature. By performing this fitting through a set range of temperatures (25–250 °C), an operationally defined glass temperature can be evaluated. More information on the fitting procedure will be given in the next section [13].

**Figure 2:**
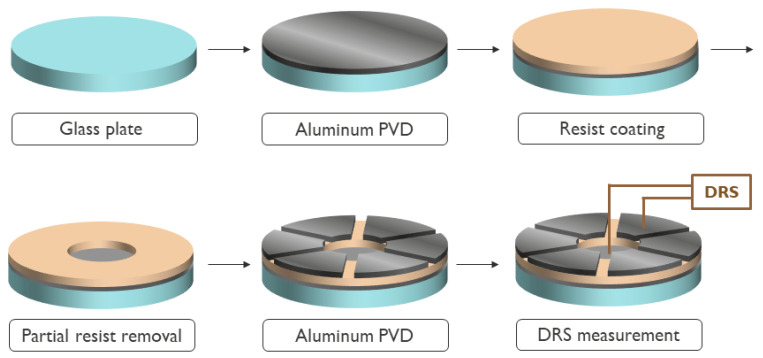
Schematic representation of a sample preparation for a dielectric response spectroscopy (DRS) measurement of a resist layer.

The samples for the DRS measurement were prepared by performing a physical vapor deposition (PVD) of aluminum (≈30 nm) onto a glass substrate, after which the targeted resist, underlayer, or both were spin coated on top of the aluminum electrode at the calibrated rounds per minute (RPM) to achieve the desired film thickness. The baking temperature for the polymers to remove the residual solvent was 90 °C for 60 s. For the underlayers, the vendor recommended settings were used. Afterwards, a small area in the middle of the sample was removed by chloroform (CHCl_3_) to enable contact with the bottom electrode. Subsequently, another PVD process through a mask deposition was performed to cap the resist with an aluminum layer to obtain the second electrode. A schematic representation of this process can be found in Figure 2. The thickness of the samples was verified by spin coating a few milliliters of the desired solution onto a silicon substrate, baking them at 90 °C for 60 s, and measuring them with ellipsometry. 

To obtain the “structural relaxation time versus temperature” plots that are used to extract the T_g_ of the sample as described above, the α-relaxation peaks of all the separate isothermal plots were fitted. Since the fitting procedure is well-known, a short description of the fitting procedure is given below; for a more in-depth explanation and example, we refer to the Supporting Information of this paper. 

The raw dielectric spectrum obtained from the measurement is subjected to a three-function fitting: (1) a function related to the conductivity of the sample, (2) the Havriliak–Negami function that fits the α-relaxation peak, and (3) a second Havriliak–Negami peak that accounts for the response of the cell. The first Havriliak–Negami fit will give the log of the frequency where the α-peak reaches its maximum ($\log\left(f_{max}\right)$). Then, this parameter can be transformed to the structural relaxation time (τ_α_), which belongs to the temperature at which the isothermal graph is fitted. This procedure is repeated for every temperature step in the range that was measured (e.g., 25–200 °C) and for which the α-relaxation peak is visible. The α-peak fitting was only accepted if the overall fit had an R^2^ > 0.99 and the parameters related to the two other peaks were stable. Then, all fits were extended by using the isochronal data to obtain more accurate low temperature data points. When all the structural relaxation times are plotted in function of the inverse of their temperature, a portion of this graph showcases a clear thermodynamical equilibrium (i.e., it shows an increased relaxation time for lower temperatures). This portion will be subject to a fit with the Vogel-Fulcher-Tammann (VFT) equation from which an operationally defined dynamic T_g_ can be evaluated at the criterion τ_a_ equals 100 s. This corresponds to a scanning rate of 10 K/min that is conventionally used to determine the thermal T_g_.

#### 2.2.3. EUV Exposure and Characterization

The sample preparation for EUV exposures was done by spin coating a commercial organic underlayer (20 nm) on a silicon wafer that was primed with a sub-nm hexamethyldisilazane (HMDS) monolayer. On top of this layer, the different resist samples were spin coated at a thickness of 25 nm and baked at 90 °C for 60 s. The wafers were subsequently exposed using a full-field NXE:3300B scanner with a custom X-dipole illumination to achieve vertical lines and spaces. Afterwards, the wafers received a post-exposure bake of 90 °C for 60 s and were developed with a 2.38% tetramethyl–ammonium hydroxide solution. Patterning images were taken with a Hitachi 6300 CDSEM, after which the roughness and CD values were obtained by unbiasing with the Fractilia MetroLER software and averaging 50 images (1600 nm × 1600 nm) per pitch for a total area of 2.6 μm^2^.

## 3. Results and Discussion

### 3.1. Influence of Platform with Different Protecting Groups on the T_g_

It is well known that several factors can influence the T_g_ of a polymer. The most important factors include the degree of polymerization, the chain rigidity, the pendant groups, and the resulting interaction of the chains with each other. As a base of comparison, the bulk T_g_ values of the three different polymers were measured by differential scanning calorimetry (DSC) and given in Table 1. The results indicate that the lowest thermal T_g_ was found for P(HS-*r*-MCPMA), followed by P(HS-*r*-tBuMA) and P(HS-*r*-MAdMA). The obtained trend can be explained through the size of the pendant group and interaction of the different polymer chains. In the case of the *tert*-butyl group, the overall size has the smallest impact on rotational energy since its size is the smallest, but the interaction with neighboring chains is large because it is relatively easy to get entangled. For the 1-methylcyclopentyl group, the overall size of the pendant group is larger, but the resulting entanglement with the neighboring chains is lower, since it is a closed cyclic group. The 1-methyladamantyl group acts more as an anchor and is very effective in restricting the movement of the polymer chains, thus resulting in the highest T_g_ value. The obtained trend is in accordance with previously reported data on the impact of pendant groups on T_g_ [14,15].

**Figure 3:**
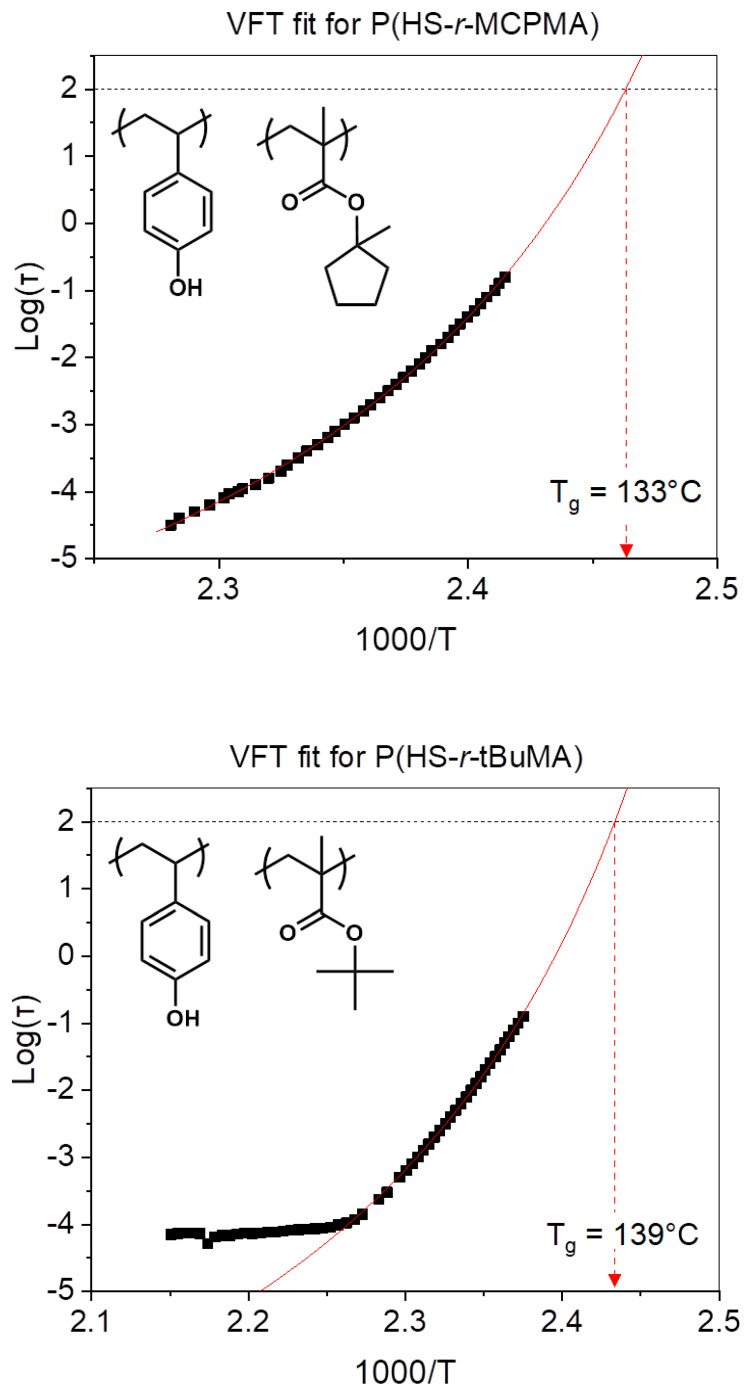

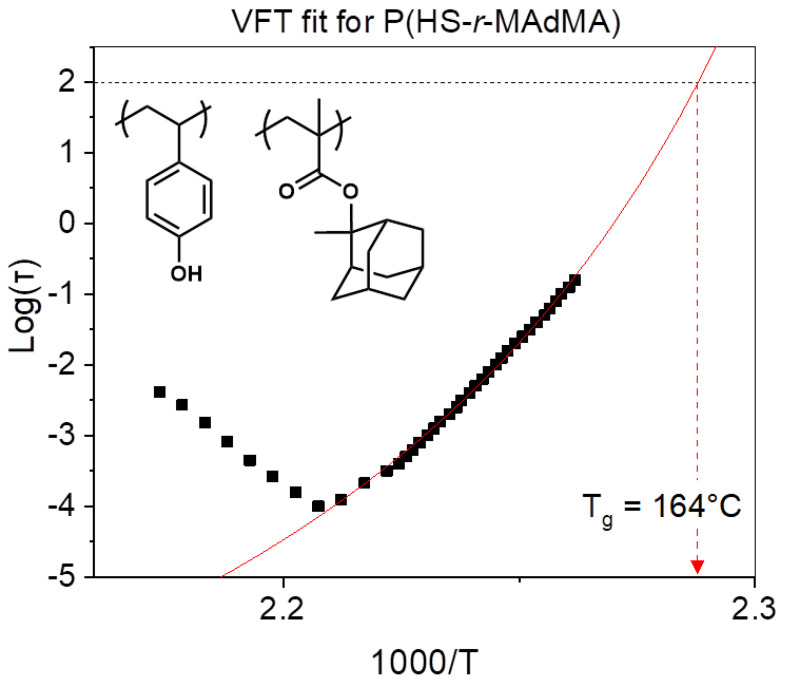
Logarithmic structural relaxation time versus the inverse of temperature plots for the different 30 nm thin film polymer samples.

By going to polymer thin films, the measurement of the bulk thermal T_g_ is not an accurate representation anymore of the actual T_g_ of the system. By measuring the dielectric response of a 30 nm film of the different P(HS-*r*-MMA) platform polymers, the impact of the pendant group on the thin film dynamic T_g_ was determined. The structural relaxation time for the different polymers in function of the temperature is given in Figure 3. As described in the fitting method in the supplementary information, the data points that are within a clear thermodynamic state were fitted with a VFT equation to obtain the T_g_. The subsequent points that deviate from the expected trend are attributed to a phase transition or immobilization of the system, but they do not influence the accuracy of the determination of T_g_ since they are outside the temperature region of interest. 

Comparing the DSC measurements to the measurements of DRS yields the same T_g_ trend, but the T_g_ values from the DRS measurements are higher compared to the bulk material. A logical reason to explain this behavior is that the interaction between the polymer chains and the substrate is stronger compared to the intermolecular interactions of the polymer chains, leading to a rise in T_g_ value of the polymer thin film. Interestingly, the increase of the T_g_ value is more severe for the lower T_g_ polymers. Since the interaction strength between the polymer strands will be different for the three polymer types, as well as their interaction with the substrate, their increase in T_g_ can be attributed to the relative difference in intermolecular interaction strength for each polymer. Observing a steeper increase for low T_g_ polymers leads to the conclusion that their inherent intermolecular interactions are much weaker compared to their polymer–substrate interaction, while the impact of a stronger polymer–substrate interaction for a high T_g_ will be limited because the intermolecular interaction is already strong.

### 3.2. Influence of Film Thickness and the T_g_

**Figure 4:**
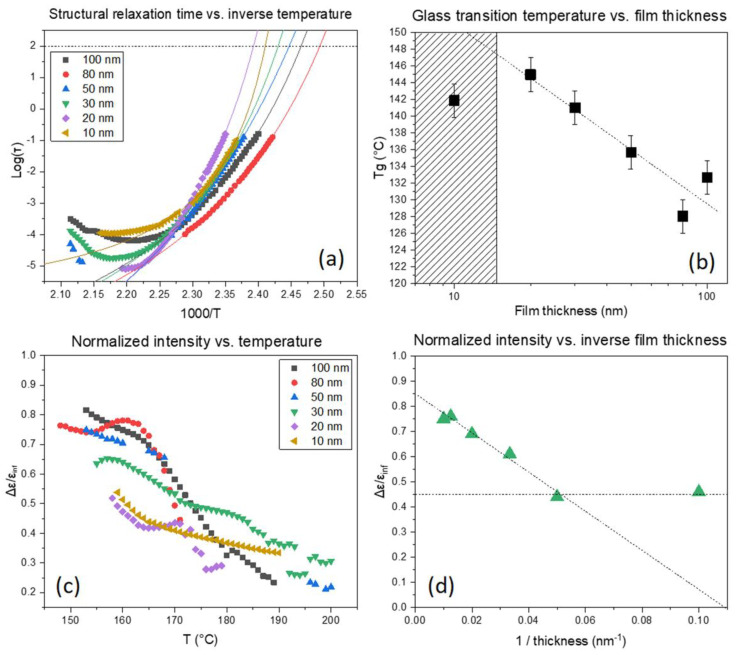
(**a**) The determined T_g_ values for each thickness; (**b**) the normalized intensity versus temperature; (**c**) and the normalized intensity versus the inverse film thickness (**d**).

Since a thickness reduction of the resist layer for high NA applications is envisioned, the influence of film thickness on the T_g_ was determined for the P(HS-*r-*tBuMA) polymer system, since this system best resembles a conventional EUV resist. The structural relaxation time for the different film thicknesses is given in Figure 4a. Again, a clear thermodynamic state is achieved within the relevant temperature range and fitted with a quadratic equation to determine the T_g_ given in Figure 4b. The trendline shows an increase in the T_g_ going to thinner films, going from about 130 to 145 °C for 100 to 20 nm polymer film thickness, and then, it shows a sudden drop in the transition temperature for the 10 nm film. This trend is also seen when the data are normalized to the intensity of the signal to exclude the effect of geometry (i.e., the contraction of the film would increase the intensity, which gives a false impression that the film is thicker due to an increase in dipole moments), as seen in Figure 4c. This trend also shows that the intensity gradually drops with decreasing film thickness because fewer dipoles react to the applied field and the same deviating result for the 10 nm film thickness. To visualize this trend more clearly, a graph that plots the thickness at a function of the intensity at a fixed temperature is given in Figure 4d. In this case, a linear trend is obtained for the increase of the T_g_ with a reduction in film thickness. It also shows that the intensity for the 10 nm sample is at the same value as the one for the 20 nm sample. This sudden change in intensity indicates that something other than just an immobilized layer is formed. A possible explanation resides in the degree of polymerization for conventional EUV CAR platforms. Since this is limited (≈7k Mw), the resulting radius of gyration is expected to be between 2 and 3 nm, which is relatively small. Therefore, the polymer itself is very sensitive to substrate and easily influenced by it. In the case of the 10 nm sample, the polymer strands at the interface are oriented differently and appear to be moving less, resulting in an intensity that is greater than expected. Essentially, somewhere between the 20 and 10 nm sample, a transition happens to a possible different stacking and/or composition layer compared to the thicker layers, which also explains the sudden change in T_g_ when the interfacial adjusted layer is reached. Moreover, this interfacial layer may also affect the homogeneity of resist additives, such as the photoacid generator (PAG) and quencher, which most certainly will affect the resist patterning performance. This sensitivity might also partially explain the differences in patterning behavior of EUV resists on different substrates [7]. 

### 3.3. Influence of Underlayers on the T_g_

**Figure 5:**
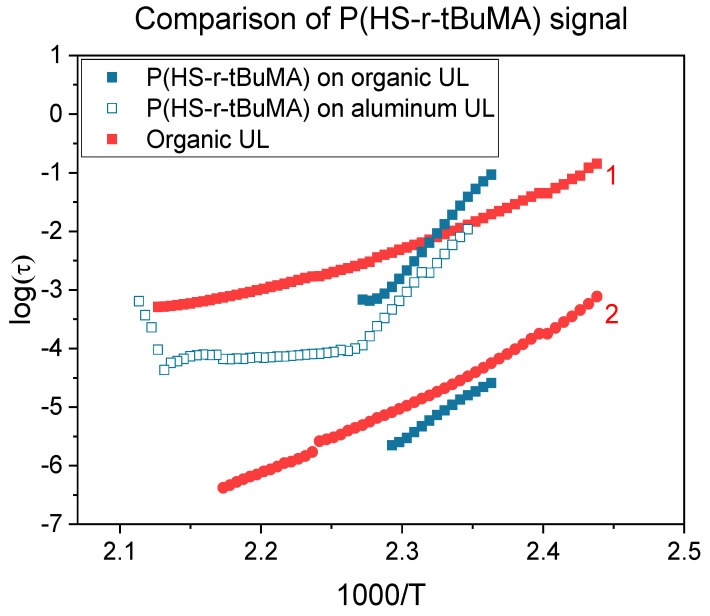
Comparison of DRS signals for the organic underlayer (red) and P(HS-*r*-tBuMA) on the organic underlayer (blue).

**Figure 6:**
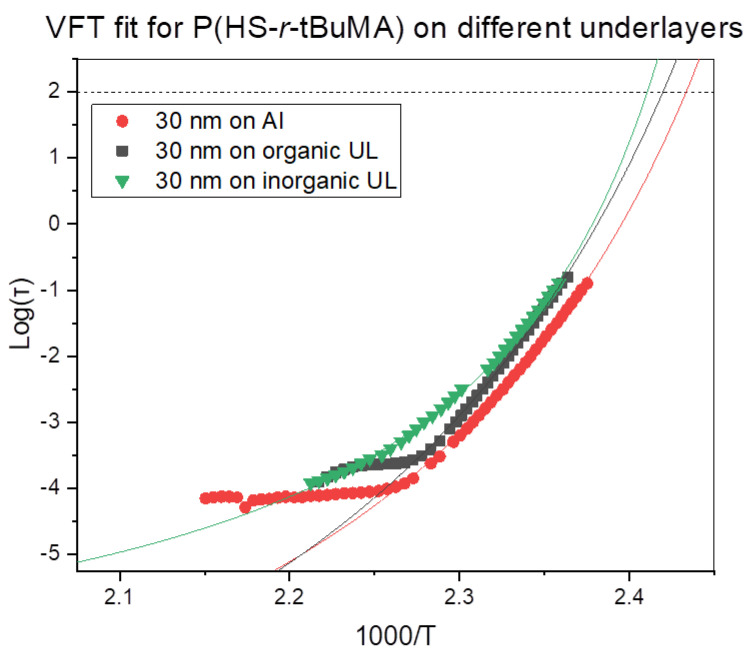
Comparison of VFT fits for P(HS-*r*-tBuMA) on the different underlayers.

**Figure 7:**
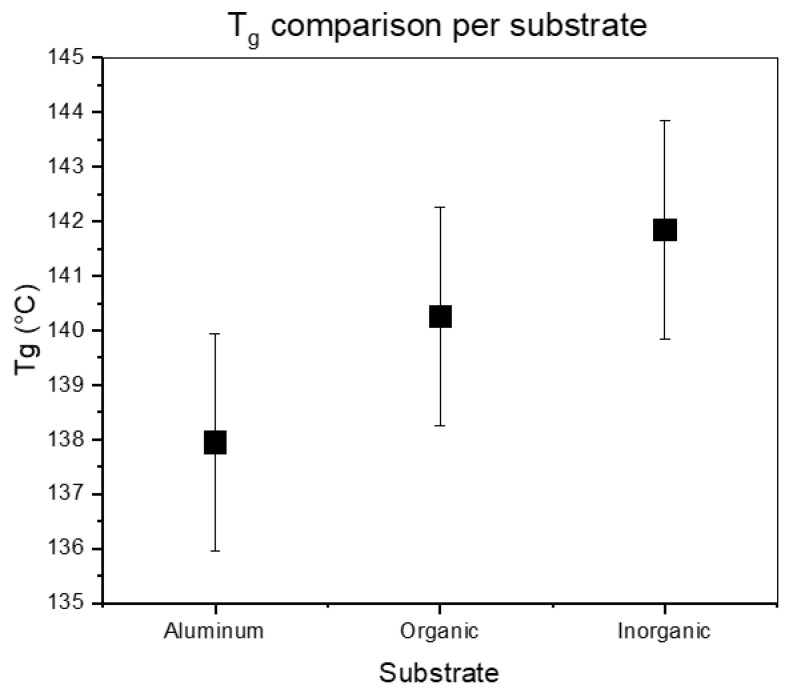
Comparison of T_g_ values on the different underlayers.

Since the previous section indicated that the polymer film was sensitive to the substrate, two relevant commercial patterning spin-on underlayers (organic and inorganic) were tested to assess the impact of different underlayers on the T_g_. To ensure an accurate determination of the T_g_ of a stack, the dielectric response of the underlayers were measured first. The organic underlayer showed two different processes, one at high (1) and one at low (2) intensity, which could have been detrimental to the determination for the polymer response as seen in Figure 5. However, when the full stack (underlayer and polymer) was measured, the higher intensity process (1) showed a clear change related to the polymer layer on the underlayer, enabling the determination of T_g_. The spin-on inorganic underlayer behaved as a resistive material under the applied field that seemed to absorb polarization, which could also trouble the measurement with the polymer layer on top. However, as with the organic layer, the resulting change in the spectrum with the full stack can be attributed to processes in the polymer film, also enabling the determination of T_g_ in this case. The VFT fits of the P(HS-*r*-tBuMA) are given in Figure 6. The summary of these measurements can be found in Figure 7 and Table 2.

Surprisingly, the results show that the underlayer has a limited effect on the increase of the T_g_ at 30 nm polymer film thickness, since the results are close to or within the range of error. To clarify these results, we propose two explanations. (1) One could remark that both the commercial organic and inorganic underlayer enable high resolution line and space pattern printing through EUVL. Since they provide similar patterning performance, the interaction between the underlayer and resist film may as well be similar. (2) It was shown in the film thickness results from Figure 4b that the largest effect is seen at the lowest tested film thickness of 10 nm. It is expected that the effect of the underlayer will be strongest at this film thickness; hence, measuring thinner resist films on the underlayers will provide more conclusive results. Measurements on these film thicknesses around 10 nm were conducted; however, the extrapolation to obtain is less straightforward due to a subdued signal. No conclusive results are available at the time of writing and will be referred to a later publication.

### 3.4. EUV Patterning Performance of the Full Resist Formulation 

**Figure 8:**
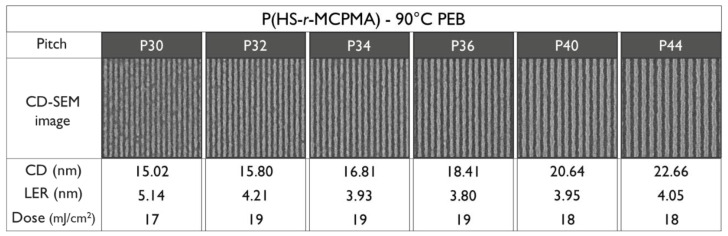
Patterning performance of the P(HS-*r*-MCPMA) polymer-based resist at 90 °C post-exposure bake (PEB).

**Figure 9:**
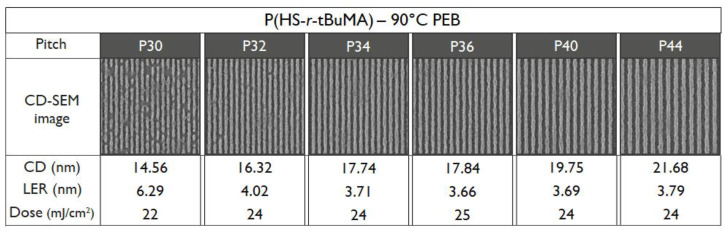
Patterning performance of the P(HS-*r*-tBuMA) polymer-based resist at 90 °C PEB.

**Figure 10:**
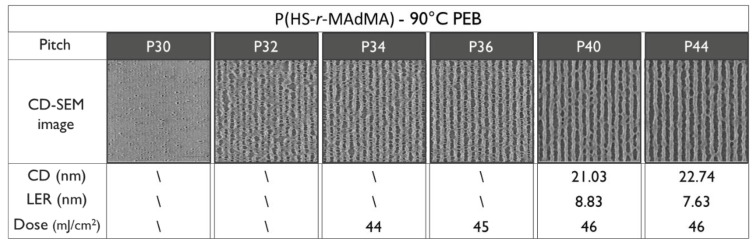
Patterning performance of the P(HS-*r*-MAdMA) polymer-based resist at 90 °C PEB.

To be able to compare the DRS results of the polymers to the patterning performance of the full resist formulation, an overview of the exposure results is given. For an initial fair comparison, it was opted to keep the PEB temperature for all the resists the same—hence, the only difference in the process conditions would be the resist that was used. The PEB temperature of 90 °C was chosen based on the best performing PEB temperature in a temperature screening done by the material supplier on the P(HS-*r*-tBuMA) resist, for which the chemical structure is closest to conventional EUV CAR systems. Not surprisingly, the results (Figure 8, Figure 9 and Figure 10) show that indeed, the P(HS-*r*-tBuMA) resist has the best patterning performance in terms of achieved line-edge roughness (LER) while having the P(HS-*r*-MCPMA) resist as a close second. The final resist P(HS-*r*-MAdMA) fails to show any fully resolved lines and spaces at the tested pitches.

The differences in patterning performance can partially be linked to the different T_g_ values of the polymers used in the resist. When using the same PEB temperature, a low T_g_-value resist will cause more acid diffusion, causing deprotection reactions into the unexposed lines. This process causes more defects (i.e., line breaks) and more deviation from the ideal line profile—hence, an increased LER value as seen in the P(HS-*r*-MCPMA) case. A high T_g_ value will limit the acid diffusion, ideally allowing some chemical amplification of the aerial image to completely resolve the lines. However, a T_g_ value that is too high will limit the acid diffusion almost completely, causing many bridging defects and a high LER value due to insufficient deprotection reactions as seen in the P(HS-*r-*MAdMA) resist. As mentioned, the PEB temperature that was used was optimized for the P(HS-*r*-tBuMA) resist where it gives the best trade-off between chemical amplification and controlling acid diffusion. An additional remark can be made regarding the dose necessary to resolve the 1:1 line and space pattern; for the same PEB temperature, the different doses necessary to resolve the pattern follows the T_g_ trend, where a low T_g_ value results in a low dose and a high T_g_ value results in a higher dose. This is also related to using the same PEB temperature—a low T_g_ resist will require less generated acids, as the acid can deprotect over a larger area due to enhanced acid diffusion. In the same way, a high T_g_ resist will require more generated acids, as it can only deprotect over a limited area due to limited acid diffusion.

**Figure 11:**
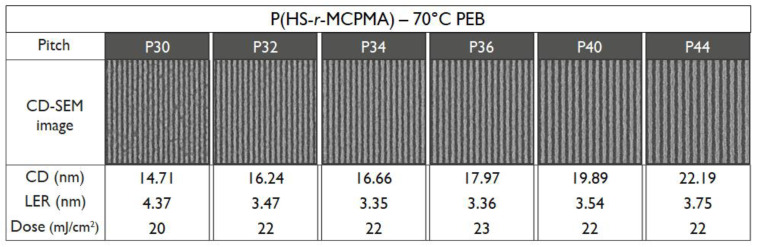
Patterning performance of the P(HS-*r*-MCPMA) polymer-based resist at 70 °C PEB.

**Figure 12:**
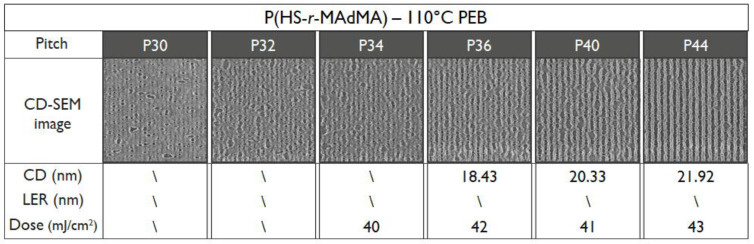
Patterning performance of the P(HS-*r*-MAdMA) polymer-based resist at 110 °C PEB.

**Figure 13:**
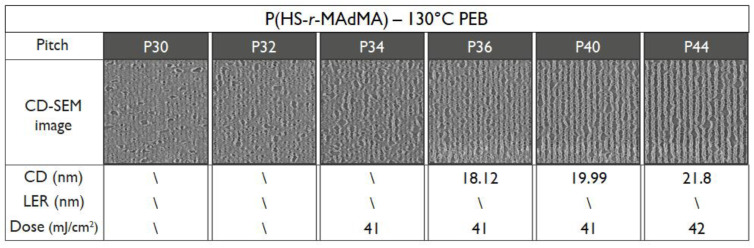
Patterning performance of the P(HS-*r*-MAdMA) polymer-based resist at 130 °C PEB.

**Figure 14:**
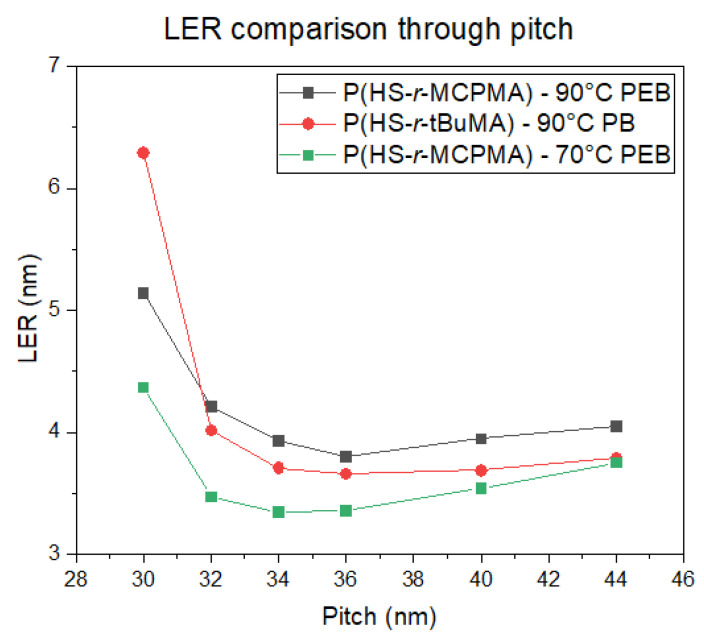
Unbiased line-edge roughness (LER) comparison between P(HS-*r*-tBuMA) and P(HS-*r*-MCPMA) resists.

To verify the T_g_ influence on the patterning performance, a lower and higher PEB temperature for the P(HS-*r*-MCPMA) and P(HS-*r*-MAdMA) resist was tested respectively. In the former case, reducing the PEB temperature to 70 °C significantly improved the LER values (Figure 11) due to a more controlled acid diffusion, making the resist perform even better than the P(HS-*r-*tBuMA) resist. In contrast, raising the PEB temperature to 110 °C (Figure 12) or even 130 °C (Figure 13) for the P(HS-*r*-MAdMA) resist did not result in an improvement of the patterning formation or LER values, indicating that, besides the influence of T_g_ on acid diffusion, more parameters such as the dissolution rate of the resist play a role. A summary of this result can be seen in Figure 14. It is also important to mention that while the T_g_ of the polymers was measured, additives such as PAG and quencher will lower the T_g_ value of the full resist. As indicated in a previous work, the T_g_-to-PEB temperature relation is an important factor in resist patterning formation. In resists where T_PEB_ > T_g_, the polymer chains will be redistributed during the PEB process due to large hydrophilic deprotected and hydrophobic protected sites. This opposed to a resist where T_PEB_ < T_g_ where the rearrangement of polymer strands did not occur and led to a higher final edge roughness of the pattern [16]. Ideally, the next step in this research would be to determine the T_g_ of the full resist formulation and relate this to the patterning performance that was observed.

## 4. Conclusions

In this study, a methodology to investigate interfacial interactions through the determination of T_g_ is given. For the experiments, three different polymers were tested, of which, one polymer was also tested with various film thicknesses and on different underlayers. By using dielectric response spectroscopy, the results obtained show that changing the protection group of the polymer, as well as altering the film polymer thickness impacts the dynamics of the polymer mobility, which can be assessed through the T_g_ of the system. By thinning down the polymer thickness, the interfacial behavior between the polymer and the underlayer become increasingly dominant. This results in an increase of the T_g_ and indicates the existence of an interfacial layer with different morphology and/or composition compared to the “bulk” polymer material. While the overall increase in T_g_ value is limited when moving from the current film thickness (≈35 nm) to the expected high NA film thickness (≈20 nm), the interfacial behavior will have to be considered for future high NA resist material design, as it may affect resist patterning performance. Examples of this could be (I) inducing chemical inhomogeneity or (II) changes in adhesion of the resist to the substrate resulting in broken lines (weak adhesion) or residues (strong adhesion) over the depth of the interfacial layer. It is important to note that the interfacial behavior will most likely become more dominant as the resist film thickness shrinks further for (improvements to) high NA EUVL or beyond. This means that it becomes important to co-optimize the underlayer together with the resist rather than just develop a good resist material that can comply with the strict goals set for resolution, line-edge roughness, and sensitivity (RLS).

As an outlook, the next step in this research would be to (I) determine the steep decrease in T_g_ for thin films more accurately and (II) determine the T_g_ of the full resist formulation and relate this to the patterning performance that was observed to obtain a direct link, rather than correlating the resist patterning performance to an obtained polymer T_g_ trend. Initial experiments show that the T_g_ determination for the full resist formulation is less straightforward, as the T_g_ is lowered, and a larger extrapolation is necessary. In this case, an alteration or optimization of experimental conditions will also be needed.

## Figures and Tables

**Table 1:** Differential scanning calorimetry (DSC) (bulk) and DRS (30 nm thin film) measurement results for T_g_ for different polymers.

Polymer	T_g_ (DSC)	T_g_ (DRS)
P(HS-*r*-MCPMA	79 °C	133 ± 2 °C
P(HS-*r*-tBuMA)	98 °C	139 ± 2 °C
P(HS-*r*-MAdMA	133 °C	164 ± 2 °C

**Table 2:** DRS (30 nm thin film) measurement results for T_g_ for different underlayers.

Underlayer	T_g_ (DRS)
Aluminum	138 ± 2 °C
Organic	140 ± 2 °C
Inorganic	142 ± 2 °C

## Supplementary Materials

The following are available online at https://www.mdpi.com/2073-4360/12/12/2971/s1, Figure S1: Example fit of a 30 nm film thickness P(HS-r-tBuMA) sample at 166°C, Figure S2: Example VFT fit of a 30 nm film thickness P(HS-r-tBuMA) sample with isothermal data, Figure S3: Example of a quadratic fit of a 30 nm film thickness P(HS-r-tBuMA) sample at 631 Hz, Figure S4: Example VFT fit of a 30 nm film thickness P(HS-r-tBuMA) sample with isochronal data, Figure S5a: Overlap of the isothermal and isochronal data fits, Figure S5b: Combined isochronal and isothermal data fit of a quadratic fit of a 30 nm film thickness P(HS-r-tBuMA) sample at 631 Hz.

## References

[1] Samsung Electronics Begins Mass Production at New EUV Manufacturing Line. https://news.samsung.com/global/samsung-electronics-begins-mass-production-at-new-euv-manufacturing-line.

[2] Halfacree G. TSMC’s EUV N7+ Node Hits Volume Production. https://bit-tech.net/news/tech/cpus/tsmcs-euv-n7-node-hits-volume-production/1/.

[3] Van Schoot J., Zahlten C., Gräupner P., Kürz P., Stoeldraijer J., Kaiser W. (2019). High-NA EUV lithography pushing the limits. Proc. SPIE.

[4] Lio A. (2016). EUV resists: What’s next?. Proc. SPIE.

[5] Chini S.F., Amirfazli A. (2010). Understanding Pattern Collapse in Photolithography Process Due to Capillary Forces. Langmuir.

[6] Silva D., De Silva A., Dutta A., Meli L., Yao Y., Mignot Y., De Silva A., Dutta A., Meli L., Yao Y. (2020). Inorganic hardmask development for EUV patterning. Proc. SPIE.

[7] Vanelderen P., De Simone D., Spampinato V., Franquet A., Vandenberghe G. (2018). The Role of Underlayers in EUVL. J. Photopolym. Sci. Technol..

[8] Jablonski E.L., Prabhu V.M., Sambasivan S., Fischer D.A., Lin E.K., Goldfarb D.L., Angelopoulos M., Ito H. (2004). Surface and bulk chemistry of chemically amplified photoresists: Segregation in thin films and environmental stability issues. Proc. SPIE.

[9] Malik S., Eisele J., Whewell A., Ferreira L., Holt T., Bowden M. (2000). Post-Exposure Bake Temperature Considerations for High Activation Energy Resist Systems. J. Photopolym. Sci. Technol..

[10] Forrest J.A., Dalnoki-Veress K., Stevens J.R., Dutcher J.R. (1996). Effect of free surfaces on the glass transition temperature of thin polymer films. Phys. Rev. Lett..

[11] Yang Z., Clough A., Lam C.H., Tsui O.K.C. (2011). Glass transition dynamics and surface mobility of entangled polystyrene films at equilibrium. Macromolecules.

[12] Callen H.B., Welton T.A. (1951). Irreversibility and Generalized Noise. Phys. Rev..

[13] Napolitano S., Glynos E., Tito N.B. (2017). Glass transition of polymers in bulk, confined geometries, and near interfaces. Reports Prog. Phys..

[14] Nakamichi T. (1995). Glass Transition Temperature and Free Volume (I). J. Jpn. Soc. Colour Mater..

[15] Uematsu I. (1957). Crystallization and Glass Transition (Second Order Transition) of Polymers. J. Jpn. Soc. Colour Mater..

[16] Park J., Lee S.-G., Vesters Y., Severi J., Kim M., De Simone D., Oh H.-K., Hur S.-M. (2019). Molecular Modeling of EUV Photoresist Revealing the Effect of Chain Conformation on Line-Edge Roughness Formation. Polymers.

