# Supplementary material for: Dielectric Response Spectroscopy as Means to Investigate Interfacial Effects for Ultra-Thin Film Polymer-Based High NA EUV Lithography
Source: Polymers (Basel). 2020 Dec 12;12(12):2971. doi: 10.3390/polym12122971 (PMC7762983; doi:10.3390/polym12122971)
Supplement: Supplementary file 1 [file polymers-12-02971-s001.pdf]

SUPPORTING INFORMATION

To obtain the "structural relaxation time versus temperature"-plots that are used to extract the T_g of the sample as described above, the α-relaxation peaks of all the separate isothermal plots need to be fitted. To fit the dielectric spectrum that is obtained, the Grafity software package is used that can combine several functions to accurately fit the spectrum. In total, three functions are used to describe the dielectric spectrum, as seen in **Figure S1**. A first function (1) is related to the conductivity of the sample and will influence the slope of the left side of the spectrum. The second function is an empirical modification of the Debeye relaxation model and is called the Havriliak-Negami equation (2) that accounts for the asymmetry of the dielectric dispersion curve, which describes the α-relaxation peak that is observed in the middle of the spectrum. The third and last function is a second Havriliak-Negami peak, which influences the right side of the spectrum and originates from the response of the cell and acts as a Debeye-peak (i.e. does not intrinsically have asymmetry of the peak).

$$\varepsilon''(f) = \frac{\sigma}{\varepsilon_0 (2\pi f)^n} \tag{1}$$

$$\varepsilon''_{HN} = \left\{ \frac{\Delta\varepsilon}{[1 + (if \,/\, f_0)^a]^b} \right\} \tag{2}$$

$$\log(\tau) = \log(1) - (\log(2\pi) + \log(f_{max}) \tag{3}$$

$$log(\tau) = A + \frac{B}{T - T_0} \tag{4}$$

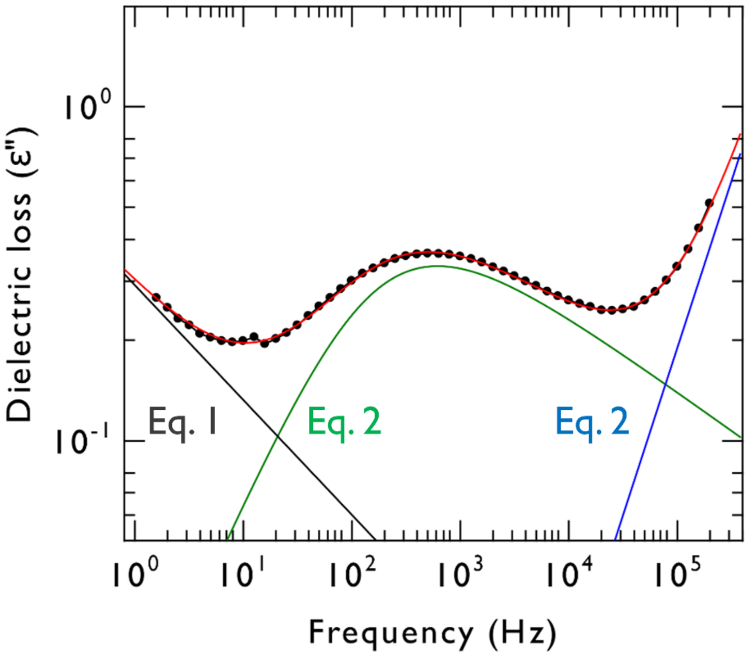

Figure S1. Example fit of a 30 nm film thickness P(HS-r-tBuMA) sample at 166°C.

By applying this fit, the log of the frequency where the α-peak reaches its maximum (log (f_{max})) is obtained. This parameter can then be transformed to the structural relaxation time (τ_α) as shown in (3). The obtained τ_α belongs to the temperature at which the isothermal graph is fitted. This procedure is repeated for every temperature step in the range that was measured (e.g. 25 – 200°C) and for which the α-relaxation peak is visible. The α-peak fitting was only accepted if the overall fit had an $R^2 > 0.99$ and the parameters related to the two other peaks were relatively stable. When all the structural relaxation times are plotted in function of the inverse of their temperature, a portion of this graph showcases a clear thermodynamical equilibrium (i.e. shows an increased relaxation time for lower temperatures). This portion will be subject to a fit with the Vogel-Fulcher-Tammann equation (4) from which an operationally defined dynamic T_g can be evaluated at the criterion τ_a equals 100 seconds. This corresponds to a scanning rate of 10 K/min that is conventionally used to determine the thermal T_g. An example of this fit is shown in **Figure S2**.

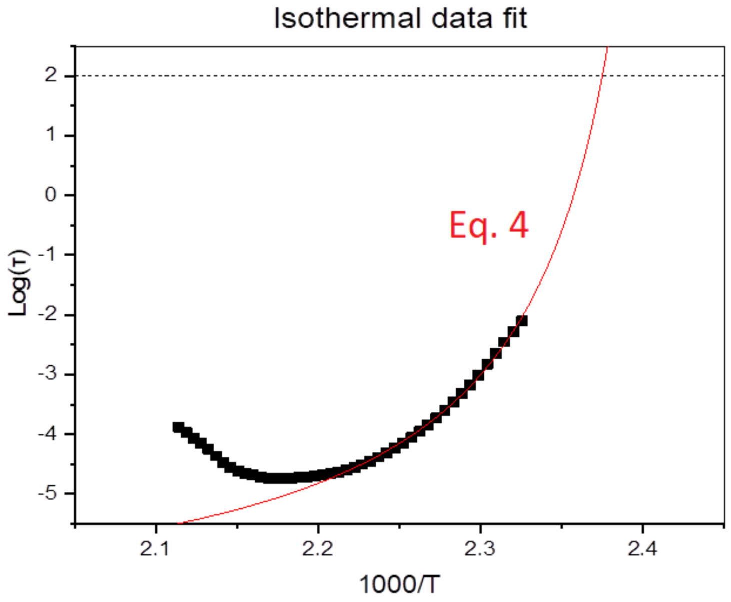

Figure S2. Example VFT fit of a 30 nm film thickness P(HS-r-tBuMA) sample with isothermal data.

However, even at an $R^2 > 0.99$ for the isothermal spectrum fit, the error becomes larger at lower temperatures because the α-relaxation peak starts to move towards the left side of the frequency window (**Figure 1**). This makes the fit less accurate because: (I) the peak mixes more with the conductivity part of the spectrum which partially masks the α-relaxation peak, and (II) it moves partially outside the available frequency window and only a portion of the α-relaxation peak can be fitted. In this case, the plot can be extended by fitting the well-defined quadratic peaks observed for lower temperatures when looking at the isochronal curves (i.e. keeping the frequency constant instead of temperature) as seen in **Figure S3**. The maximum of the quadratic fit will be a "dielectric loss – temperature" data point that can also be plotted in the same way compared to the isothermal plots and fitted to obtain a glass transition temperature (**Figure S4**). In an ideal case, a certain overlap exists between isothermal and isochronal curves (**Figure S5a**), where isochronal datapoints at low temperature are more reliable and used to extend the plot to obtain a more accurate fitting because this influences the curvature of the graph. To get a more systematic approach, points in the isothermal graph for lower temperatures were omitted until 5 points overlapped with the isochronal plot, in which case these 5 points were kept and extended with the rest of the isochronal datapoints. An example of this outcome can be seen in **Figure S5b**.

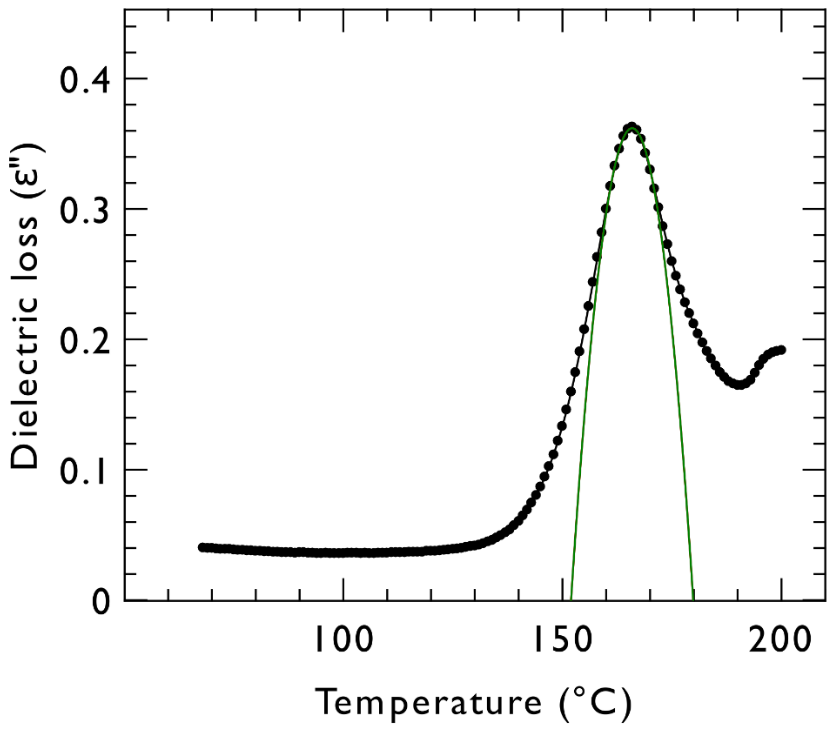

Figure S3. Example of a quadratic fit of a 30 nm film thickness P(HS-r-tBuMA) sample at 631 Hz.

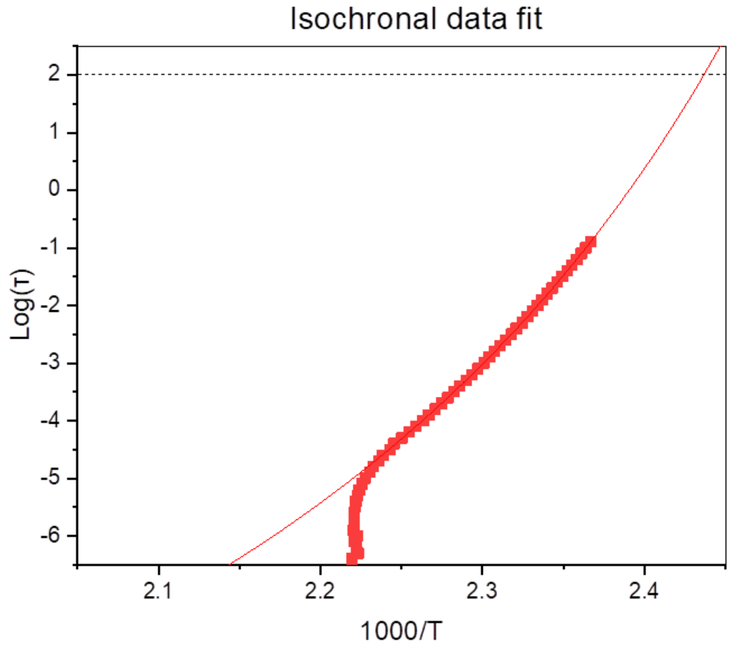

Figure S4. Example VFT fit of a 30 nm film thickness P(HS-r-tBuMA) sample with isochronal data.

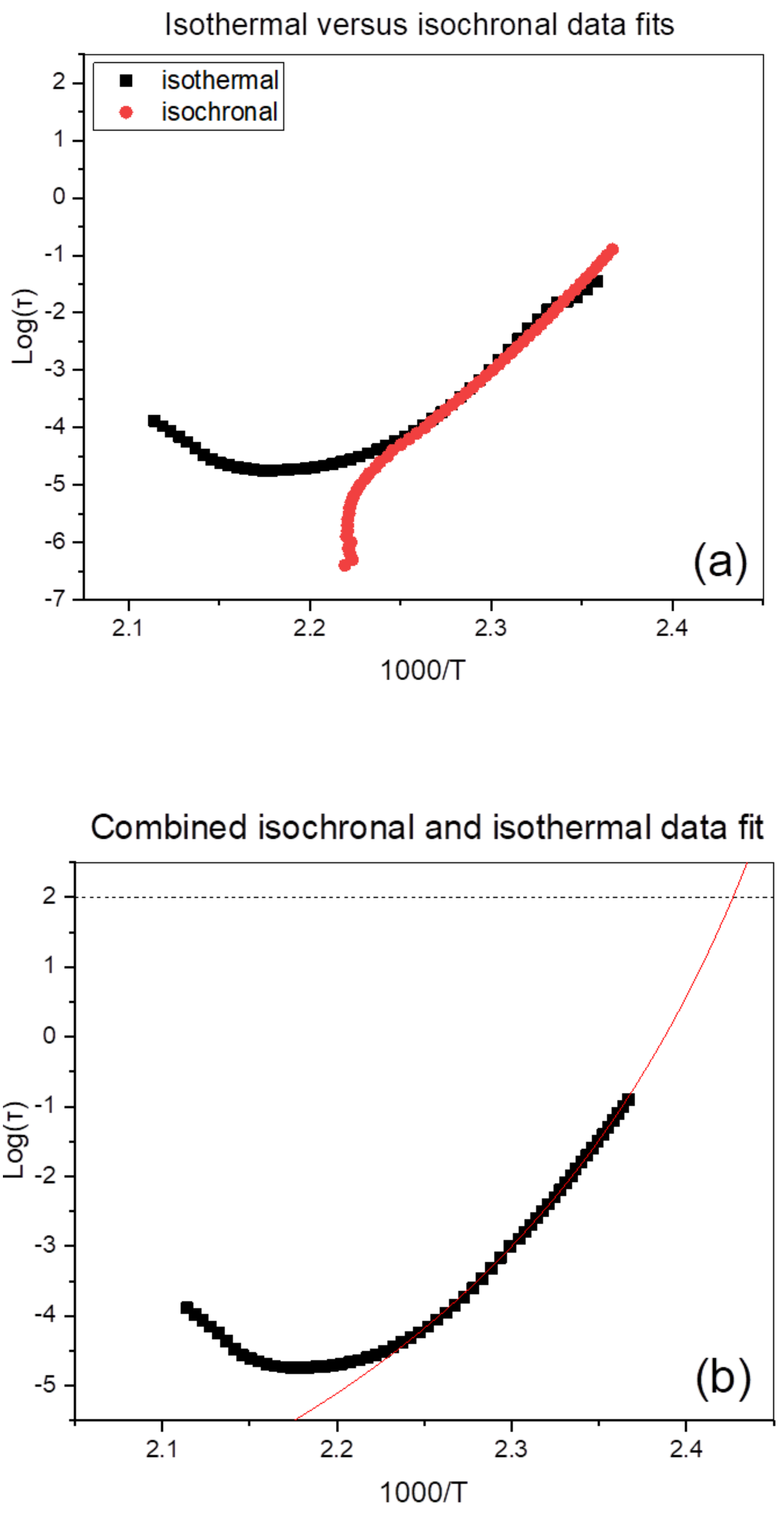

Figure S5. a) Overlap of the isothermal and isochronal data fits, b) combined isochronal and isothermal data fit of a quadratic fit of a 30 nm film thickness P(HS-r-tBuMA) sample at 631 Hz.
